# Synthesis and Characterization of Highly Intercalated Graphite Bisulfate

**DOI:** 10.1186/s11671-017-1930-2

**Published:** 2017-03-06

**Authors:** Marcella Salvatore, Gianfranco Carotenuto, Sergio De Nicola, Carlo Camerlingo, Veronica Ambrogi, Cosimo Carfagna

**Affiliations:** 1 CNR-IPCB, Institute for Polymers, Composites and Biomaterials, P.le E. Fermi, 1, Portici, 80055 Italy; 2 Department of Chemical, Materials and Production Engineering, University of Naples Federico II, P.le Tecchio, 80, Naples, 80125 Italy; 3 CNR-SPIN, Institute for Superconductors, Innovative Materials and Devices, S.S. Napoli, Complesso Universitario di M.S. Angelo, Via Cinthia, Naples, 80126 Italy

**Keywords:** Graphite intercalation compounds, Graphite bisulfate, Nano graphite, Micro-Raman spectroscopy, Fourier transform IR spectroscopy

## Abstract

Different chemical formulations for the synthesis of highly intercalated graphite bisulfate have been tested. In particular, nitric acid, potassium nitrate, potassium dichromate, potassium permanganate, sodium periodate, sodium chlorate, and hydrogen peroxide have been used in this synthesis scheme as the auxiliary reagent (oxidizing agent). In order to evaluate the presence of delamination, and pre-expansion phenomena, and the achieved intercalation degree in the prepared samples, the obtained graphite intercalation compounds have been characterized by scanning electron microscopy (SEM), energy-dispersive X-ray spectroscopy (EDS), X-ray powder diffraction (XRD), infrared spectroscopy (FT-IR), micro-Raman spectroscopy (*μ*-RS), and thermal analysis (TGA). Delamination and pre-expansion phenomena were observed only for nitric acid, sodium chlorate, and hydrogen peroxide, while the presence of strong oxidizers (KMnO_4_, K_2_Cr_2_O_7_) led to stable graphite intercalation compounds. The largest content of intercalated bisulfate is achieved in the intercalated compounds obtained from NaIO_4_ and NaClO_3_.

## Background

Graphite intercalation compounds (GICs) are technologically useful functional materials made of graphite flakes uniformly embedding small molecules or metal ions between the graphene sheets [1–5]. Such materials have been intensively studied because of the “staging phenomenon” [6] and the manifold anomalous physico-chemical behaviors [3–12]. In addition, GICs are very important in graphite manufacturing [13] and potentially useful in other industrial fields like that of superconductors [14, 15], heterogeneous catalysts [16], anode materials [17], and pyrophoric reactants stabilization [17]. Currently, GICs are used in the preparation of expandable graphite, graphite nanoplatelets (GNP), and single-layer graphene [18–21]. Graphite nitrate and graphite bisulfate have the ability to significantly expand by thermal heating. Owing to the intercalation/expansion processes, the *π*- *π* interactions, acting along the graphite c-crystallographic direction, are significantly weakened. Therefore, graphite fragmentations in separated nanocrystals take place by simply applying some sono-acustic energy. The resulting graphite nanocrystal thickness is depending on the GICs intercalation degree, usually classified in terms of “staging” index *m*, i.e., the number of graphite layers between two intercalant layers [2]; and consequently, the highest “staging index” is required in the graphene preparation. Depending on nature of the intercalating agent, the type of oxidant, and the experimental conditions, it is possible to obtain GICs as “stage I”, “stage II”, and “stage III” [14]. In particular, in a stage I compound, a single layer of graphene is alternated regularly with intercalated species. In a second stage (third stage, etc.), compound with two (three, etc.) graphene layers are separated by layers of intercalation [22]. According to the literature [23, 24], expandable graphite can be prepared by using various types of GICs hosting Brønsted acids (e.g., sulfuric acid, nitric acid, and acetic acid), metal chlorides (FeCl_3_, CuCl_3_, and ZnCl_2_) [25], gold [26], metals (Na), and alkali metals (Li, Cs, and K) in tetrahydrofuran (THF) [27, 28]. Firstly, C. Schafhäutl [29] and B. Brodie in 1851 [30] described the graphite bisulfate synthesis based on the following reaction scheme [23]:

1 $$ \begin{aligned} 24n\text{C} &+ {\text{O}_{x}}^{z} + m\mathrm{H}_{2}\text{SO}_{4}\rightarrow {{\mathrm{C}_{24}}^{+}}\cdot {\text{HSO}_{4}}^{-}\\ &\times(m-1) \mathrm{H}_{2}\text{SO}_{4} + {\text{HO}_{x}}^{\left(z-1\right)} \end{aligned}  $$

where O _*x*_ is the oxidizing agent and C is the carbon atoms in the graphite. Therefore, graphite bisulfate compounds consist of graphite layers intercalated by HSO$_{4}^{-}$ and H_2_SO_4_ molecules [23]. The stage and kinetics of bisulfate formation depend on the sulfuric acid concentration and on the type of oxidizing agent involved in the reactive system [23, 31]. At that time, very limited structural information were given in the literature concerning these systems, since X-ray diffraction was one of the few available characterization approaches. Here, graphite bisulfate has been prepared by classical liquid-phase synthesis techniques and some new reaction scheme based on never investigated oxidizing agents. The achieved materials have been fully characterized regarding their morphology and structure by scanning electron microscopy (SEM), energy-dispersive X-ray spectroscopy (EDS), X-ray powder diffraction (XRD), infrared spectroscopy (FT-IR), micro-Raman spectroscopy (*μ*-RS), and thermogravimetric analysis (TGA).

## Methods

The different graphite bisulfates were synthesized by treating graphite flakes with an oxidizing agent/sulfuric acid mixture (see Table 1). In particular, a glass flask placed in a thermostatic bath was used as reactor. Slow air-bubbling was applied to homogenize the system during the reaction. The reaction time was 1 h, and a 9:1 by volume H_2_SO_4_/oxidizing agent ratio was selected for all compositions. Such a small amount of oxidizing agent is enough to induce intercalation. The reactions were performed in isothermal condition at the temperatures indicated in Table 1. Constant quantities of graphite flakes (2 g, Aldrich, > 100 mesh), sulfuric acid (40 ml), and oxidizing agent molar amounts were used. Cold deionized water was added to reactive mixture to end the reaction. The selected temperatures were different since oxidizers have a different reactivity.

**Table 1:** Oxidizing agent used in the reactive mixtures with H_2_SO_4_ and experimental conditions

Agent	HNO_3_	KNO _3_	H_2_O_2_	KMnO_4_
Temperature reaction (°C)	40	30	40	30
Agent	K_2_Cr_2_O_7_	NaIO_4_	NaClO_3_	
Temperature reaction (°C)	30	40	30	

FT-IR and micro-Raman (*μ*-RS) spectroscopies were used to analyze the chemical structure of pure graphite and “as prepared” graphite bisulfates. FT-IR were recorded with a PerkinElmer Frontier FT-IR spectrometer, in the range 4000–800 cm ^−1^ with 4 cm ^−1^ resolution and 6 scans. The KBr pressed disc technique (1 mg of sample and 160 mg of KBr) was used. The KBr was first heated in a furnace overnight at 120 °C to minimize the amount of the adsorbed water. A Jobin-Yvon system from Horiba ISA, with a Triax 180 monochromator, equipped with a liquid nitrogen cooled charge-coupled detector was used for the *μ*-RS measurements. The grating of 1800 grooves/mm allows a final spectral resolution of 4 cm ^−1^. The spectra were recorded in air at room temperature using a 17 mW He-Ne laser source (wavelength 632.8 nm). The spectrum accumulation time was 120 s. The laser light was focused to a 2 *μ*m spot size on the sample through an Olympus microscope with ×100 optical objective. The spectra obtained were analyzed in terms of convoluted Lorentzian functions by using a best-fitting routine of GRAMS/AI (2001, Thermo Electron) program, which is based on the Levenberg-Marquardt nonlinear least-square methods. Wide-angle X-ray powder diffraction (XRD) measurements were performed using a Philips XPW diffractometer with Cu K *α* radiation (1.542 Å) filtered by nickel. The scanning rate was 0.02°/s, and the scanning angle was from 5 to 45°. A SEM (Philips model XL20) was used to investigate the morphology. EDS analysis (model Inca Oxford 250) was carried out to confirm the presence of the intercalating agent between graphite layers. The thermal expansion threshold of the different intercalation compounds was measured by thermogravimetric analysis (TGA), using a TA Q5000 instrument equipped with an infrared furnace. TGA measurements were performed using about 1.0/1.8 mg of the sample inserted in an alumina crucible placed in a platinum pan. We adopted such method in order to prevent the leakage of the sample during the expansion phenomenon. The experiments were performed under fluxing nitrogen (flow rate of 25 mL/min).

## Results and Discussion

**Fig. 1:**
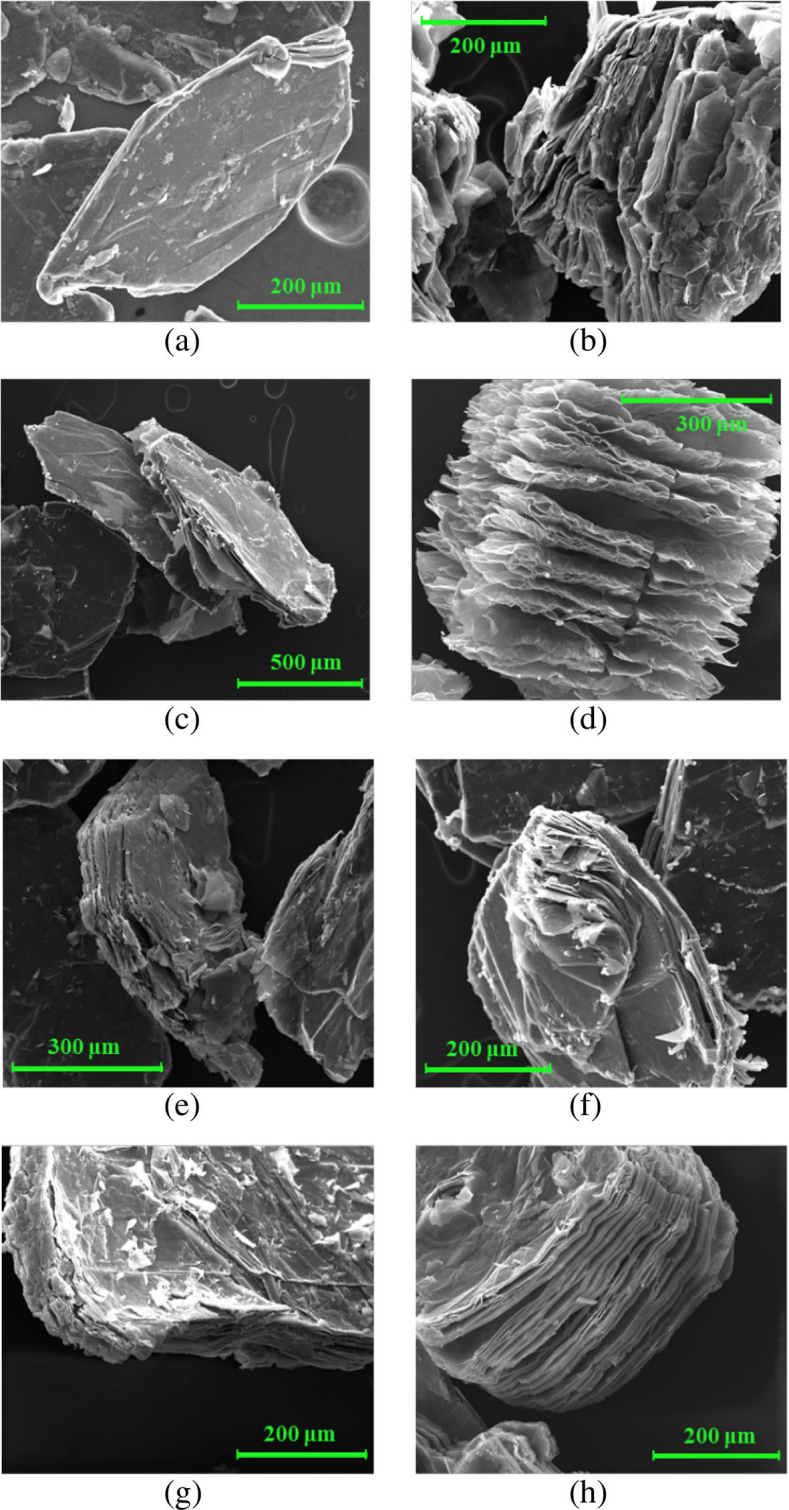
Scanning electron micrographs (SEM) of **a** starting graphite flakes, GIC from **b** HNO_3_, **c** KNO _3_, **d** H_2_O_2_, **e** KMnO_4_, **f** K_2_Cr_2_O_7_, **g** NaIO_4_, and **h** NaClO_3_

**Fig. 2:**
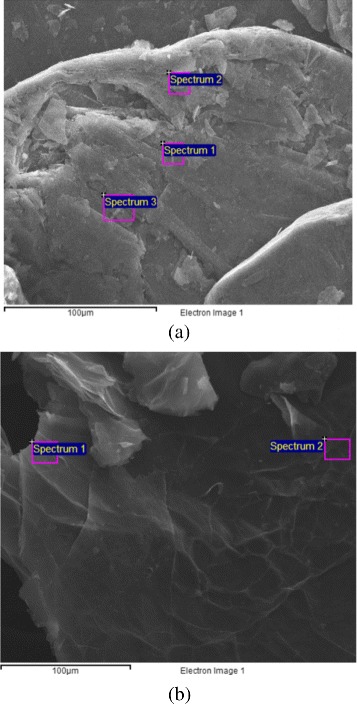
Positions where EDS analysis has been performed on **a** starting graphite flakes and **b** GIC from HNO_3_. The elemental composition is reported in Table 2

The morphology of graphite flakes after the oxidation/intercalation treatment has been investigated by comparing SEM micrographs of different GICs to that of starting graphite flakes. The obtained images are reported in Fig. 1. Treatments with H_2_SO_4_/oxidant lead to intercalated graphite. In addition to the erosion phenomenon also a delamination and pre-expansion were clearly visible for some samples. The image of a natural graphite single flake, reported in Fig. 1a, shows that the flake layers are really close to each other, and the surface is flat and uniform. Figures 1 b, f show the GICs obtained using HNO_3_ and K_2_Cr_2_O_7_ as oxidizer agent. From these images, in addition to intercalation, it is possible to observe a “delamination” phenomenon that is probably due to the strong oxidation effect. When KNO_3_ is used as an oxidizer (Fig. 1 c), only a light intercalation phenomenon occur. In this case, small white particles are observed on the intercalated flakes, probably resulting from the KNO_3_ crystals not dissolved during the chemical treatment. Furthermore, Fig. 1 d shows an image of the GIC resulting from the reaction with H_2_O_2_ as oxidizing agent. In this case, a graphite pre-expansion phenomenon occurred during the intercalation process. Such a phenomenon is probably related to the H_2_O_2_ decomposition to H_2_O and O_2_ which takes place at room temperature. Figure 1 e, g show the images of GICs obtained from KMnO_4_ and NaIO_4_, respectively. In this case, it is possible to observe the erosion of the flake boundary. This morphology is tightly connected with the intercalation process although a layer separation is not evident as in the cases of the other samples. The GIC obtained using NaClO_3_ as an oxidizing agent, Fig. 1 h, shows a strong intercalation phenomenon. From this image, it is possible to recognize multiple layers forming the flake. EDS microanalysis was carried out on small sample areas to determine nature and percentage of the elements present in the flake. As an example, we report the data of EDS spectrum from graphite and GIC obtained by HNO_3_. In Fig. 2 a, b are indicated the positions where EDS analysis has been performed on the graphite and on the GIC (HNO_3_) surface, respectively. The elemental composition for each EDS spectrum is reported in Table 2. In natural graphite, it can be clearly seen only the presence of the element carbon (besides some impurities). The EDS spectra of the other GIC samples detected a significant quantity of sulfur and oxygen due to the oxidation/intercalation process.

**Table 2:** EDS analysis: elemental composition. Results in weight %

	Spectrum	C	O	S	Si	Other	Total
	Spectrum 1	100.00	–	–	–	–	100.00
Graphite	Spectrum 2	99.50	–	–	0.50	–	100.00
(Fig. 2 a)	Spectrum 3	99.56	–	–	0.44	–	100.00
GIC (HNO_3_)	Spectrum 1	75.48	21.83	2.32	0.07	0.30	100.00
(Fig. 2 b)	Spectrum 2	70.37	21.46	7.93	0.07	0.24	100.00

**Fig. 3:**
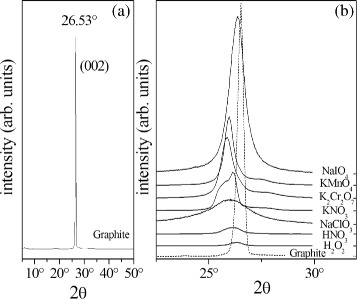
XRD of graphite and GICs. XRD (002) peak of pure graphite (**a**) and GICs (**b**).The curves in **b** are shifted arbitrarily along the *y*-axis

**Fig. 4:**
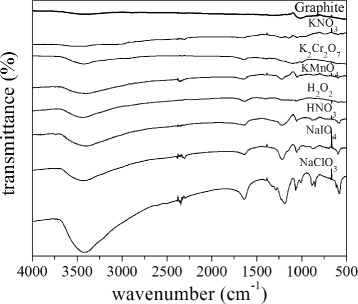
FT-IR spectroscopy. FT-IR spectra of pure graphite and GICs. The spectra are shifted arbitrarily along the *y*-axis

**Fig. 5:**
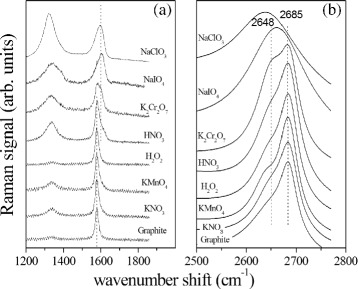
*μ*-RS of GICs in the spectral range of 1200–1850 cm ^−1^ (**a**) and 2500–2800 cm ^−1^ (**b**). The spectra are shifted arbitrarily along the *y*-axis. The *dotted lines*indicate the spectral position of the main components observed for G (**a**) and 2D mode (**b**), respectively

The structural characteristics of the obtained GICs can be evaluated by comparing XRD diffractograms of pure graphite and GICs. In Fig. 3 a, we report the diffractogram of pure graphite, and in Fig. 3 b, a portion of the same diffractogram compared with those of the GICs. As visible, the signal of GICs is significantly different from that of natural graphite. The (002) signal, which is the only one visible in the XRD pattern of natural graphite, represents reflections in the perpendicular direction (*c*-axis) of the graphite hexagonal planes. The (002) peak in the GICs X-ray diffractograms is clearly broadened compared to that of the pure graphite. Furthermore, the (002) peak results shifted to lower angles for all studied GICs compared to the pure graphite case. This result is probably due to the presence of defects in the GICs crystal lattice. This effect depends on the obtained intercalation and on the kind of oxidizing agents used in the reaction. In particular, the X-ray diffractograms of GICs obtained using NaClO_3_, KNO_3_, and K_2_Cr_2_O_7_ as oxidizing agents show the maximum 2*θ* shift of the (002) peak. The XRD spectra of GICs by HNO_3_ and H_2_O_2_ exhibit maximum broadening of the (002) peak. As it can be seen from the corresponding SEM images (Fig. 1 b, d), the samples appear to be better expanded compared to the other GICs. We ascribe the better expansion to the instability of the HNO_3_ and H_2_O_2_ oxidants which results in gas formation during the intercalation reactions. The oxidation of GIC samples was investigated by FT-IR spectroscopy. Fig. 4 shows FT-IR spectra of prepared samples; as visible, the GIC spectra are remarkably different from that of natural graphite. They exhibit an oxidation signal due to the presence of hydroxyl groups (−OH) at 3500 cm ^−1^. The natural graphite also presents a light oxidation. The oxidation degree was depending on the type of oxidizer used and has a maximum for NaClO_3_ and a minimum for the KNO_3_ oxidizer. All spectra also featured a clear signal at 1650 cm ^−1^ ascribed to the C=C double bond stretching vibration and C–O bond stretching vibration at 1200 cm ^−1^. The *μ*-RS spectra obtained from the graphite intercalated compounds are reported in Fig. 5 and compared with spectrum of pure graphite. In Fig. 5 a, the spectrum region in the wavenumber range of 1200–1850 cm ^−1^ is considered, where a prominent peak (designated as G mode) is generated by graphite at about 1582 cm ^−1^. This peak is clearly visible in the spectrum of graphite, reported in the low part of the figure, and in all other spectra, even if evident differences occur in the observed Raman response. The spectra in Fig. 5 are sorted in increasing degree of differentiation with respect to that of graphite. The spectra of the samples obtained from KNO_3_, KMnO_4_, and H_2_O_2_ look rather similar to that of graphite even if the higher intensity of Raman mode visible at about 1332 cm ^−1^ (D mode) manifests the occurrence of a relatively large amount of defects in the lattice [32]. For the samples obtained from KNO_3_, H_2_O_2_, and KMnO_4_, the Raman G mode can be successfully fitted by a single Lorentzian function centered at about 1580 cm ^−1^. This is not the case of the other spectra (related to K_2_Cr_2_O_7_, HNO_3_, NaIO_4_, and NaClO_3_), where the fit require two distinct components, represented by Lorentzian functions centered at about 1580 cm ^−1^ and 1600 cm ^−1^, respectively. Two dotted lines indicate these positions in Fig. 5 a. The first component has a center value close to the G mode position of pristine graphite and is assigned to block of not intercalated graphite layers, while the 1600 cm ^−1^ mode is assigned to graphene layers next to an intercalant layer. The ratio of the intensity of this second component with respect to the intensity of 1580 cm ^−1^ mode, evaluated by the fit procedure, gradually increases from the value of 0.32 for sample K_2_Cr_2_O_7_ to the value of 1.33 of sample NaClO_3_, indicating a significant intercalation degree of the samples, even if in different stage configurations.

For these compounds, the intensity of the D mode (at 1332 cm ^−1^) is also clearly observable in the Raman spectra (see Fig. 5 a), and it is relatively larger than in the case of GICs before considered (KNO_3_, KMnO_4_, and H_2_O_2_), indicating the occurrence of structural defects. This is also evidenced by the slight increase of both the position and the width of the G peak, that is a signature of increasing lattice defects [33]. It is worth to note that in the case of doping-like defects the increase of position of G mode with impurity degree is instead accompanied by an increasing stiffness [33].

**Fig. 6:**
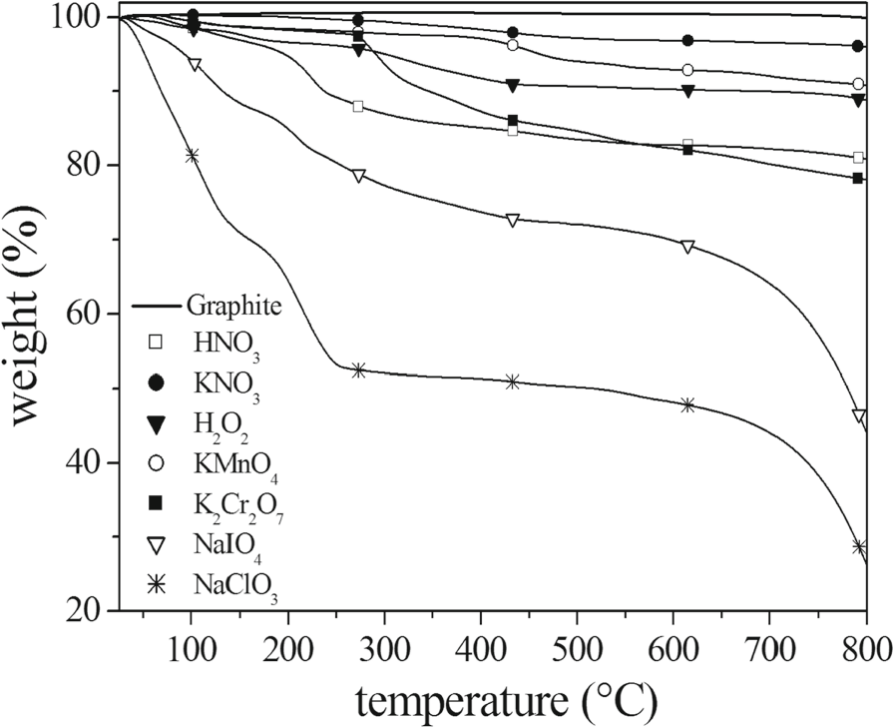
TGA results for graphite and graphite intercalated compounds

This general behavior is confirmed by the Raman spectra in the wavenumber range of 2500–2800 cm ^−1^, reported in Fig. 5 b. In this range, graphite is characterized by a broad peak (designed as 2D mode) centered at about 2685 cm ^−1^. Differently from the G mode, the position of this peak depends on the laser excitation wavelength *λ* (in our case *λ* = 632.8 nm) [33–35]. The differences with respect to graphite become gradually more significant when spectra of the sample obtained from KNO_3_ to that one derived from NaClO_3_ are considered, in the order indicated in Fig. 5 b. Beyond the pristine graphite 2D mode (at 2685 cm ^−1^), the fit of the experimental data requires an additional component at about 2648 cm ^−1^ with increasing intensity. This mode becomes the prevalent one in the samples obtained by using NaIO_4_ and NaClO_3_ as oxidizers. Two dotted lines represent the position of these two modes in Fig. 5 b. The observed shift of the 2D mode position to lower wavenumber values with respect to pristine graphite is a signature of presence of graphene sheets, indicating the formation of low stage GICs [19]. A large intercalation configuration in the NaIO_4_ and NaClO_3_ systems is inferred because the Raman signal results generated by almost monolayers of graphene (low stage number). In the remaining samples, intercalation process is also obtained but with relative thicker layer blocks of graphite (high stage number) interposed to intercalated layers. The thermogravimetric behavior of different GICs was evaluated by thermogravimetric analysis (TGA) (Fig. 6). TGA runs from 25 to 800 °C at heating rate of 10 °C min ^−1^. During the thermal treatment, an expansion phenomenon of GICs occur [13]. Intercalating agent and graphite react according to the following scheme [36, 37].

2 $$ \mathrm{C} + 2\mathrm{H}_{2}\text{SO}_{4}\rightarrow \text{CO}_{2} + 2\text{SO}_{2} + 2\mathrm{H}_{2}\mathrm{O}  $$

According to this scheme, there is a release of gases during the GIC’s expansion: carbon dioxide, sulfur dioxide, and water vapor. The weight loss, due to the gas released, can be clearly seen for all the considered samples. The weight loss ranged from 3%, for KNO_3_, up to 52% for NaClO_3_ (Table 3). The order of data in Table 3, in increasing degree of weight loss, is the same as that used to tabulate *μ*-RS results in Fig. 5, and it clearly indicates that highest intercalation degree corresponds to higher weight loss.

**Table 3:** Weight loss percentage of pure graphite flakes and GICs

Sample	Weight loss (%)
Natural graphite	0
KNO_3_	3
KMnO_4_	7
H_2_O_2_	10
HNO_3_	17
K_2_Cr_2_O_7_	18
NaIO_4_	30
NaClO_3_	52

## Conclusions

We have presented and described new chemical routes for synthetizing highly intercalated graphite bisulfate. The reaction schemes use different auxiliary reagents (oxidizing agent): nitric acid, potassium nitrate, potassium dichromate, potassium permanganate, sodium periodate, sodium chlorate, and hydrogen peroxide. Micro-Raman spectroscopy analysis of products has shown that samples treated by NaIO_4_ and NaClO_3_ lead to final products with the highest intercalation degree, which is consistent with the weight loss from the TGA data. Furthermore, according to FT-IR data, OH is the only oxygen-containing group generated during the intercalation process. EDS elemental analysis allowed to assess the presence of sulfur and oxygen characterizing the oxidation/intercalation process. The SEM micrographs, FT-IR analysis, micro-Raman spectroscopy, and calculated weight loss (from TGA analysis) confirm that the GIC, obtained using KNO_3_ as oxidizer, is the most similar to natural graphite, whereas GIC based on NaClO_3_ seems to have a higher degree of intercalation.

## Abbreviations

EDS: Energy-dispersive X-ray spectroscopy
FT-IR: Fourier transform infrared (spectroscopy)
GIC: Graphite intercalated compound
SEM: Scansion electron microscope
TGA: Thermogravimetric analysis
XRD: X-ray powder diffraction
*μ*-RS: micro-Raman spectroscopy
